# Primary Pulmonary Plasmacytoma with Diffuse Alveolar Consolidation: A Case Report

**DOI:** 10.4061/2010/463465

**Published:** 2010-06-13

**Authors:** Zohreh Mohammad Taheri, Forouzan Mohammadi, Mehrdad Karbasi, Leila Seyfollahi, Shahram Kahkoei, Mojtaba Ghadiany, Nader Fayazi, Davood Mansouri

**Affiliations:** ^1^Department of Pathology, National Research Institute of Tuberculosis and Lung Disease, Tehran 19569, Iran; ^2^Department of Radiology, National Research Institute of Tuberculosis and Lung Disease, Tehran 19569, Iran; ^3^Department of Onchology, Taleghani Hospital, Tehran 19569, Iran; ^4^Department of Pulmonology, National Research Institute of Tuberculosis and Lung Disease, Tehran 19569, Iran; ^5^Department of Infectious Disease, National Research Institute of Tuberculosis and Lung Disease, Tehran 19569, Iran

## Abstract

Solitary extramedullary plasmacytomas are plasma cell tumors that tend to develop in mucosa-associated lymphoid tissues including the sinonasal or nasopharyngeal regions. Primary plasmacytoma of the lung is exceedingly rare and often presents as a solitary mass or nodule in mid-lung or hilar areas and diagnosed after resection. Herein, we report a case of primary pulmonary plasmacytoma that presented with diffuse alveolar consolidation and diagnosed by transbronchial lung biopsy.

## 1. Introduction

Extramedullary plasmacytoma (EMP) comprises roughly 3%–5% of all plasma cell neoplasms. Eighty percent of EMP occurs in the head and neck and most cases involve the upper aerodigestive tract [1]. Primary pulmonary plasmacytoma (PPP) is a rare type of extramedullary plasmacytoma and usually present with a nodule or mass in hilar areas. Diffuse alveolar consolidation is exceedingly rare and reported in two cases [2, 3]. Here we report a case of PPP with unusual presentation.

## 2. Clinical History

A 60-year-old nonsmoker female with a chief complaint of dyspnea (function class III to IV) from three days before referred to our center. She had a 6-month history of dry cough which changed to purulent productive cough 20 days prior to admission, in addition to solid meal dysphagia, fever, chills, sweating, weight loss, and loss of appetite.

She was hospitalized with a diagnosis of unspecified pneumonia. She had a history of coronary artery angioplasty eight months ago and vague history of rheumatoid arthritis. She used corticosteroids, atorvastatin, verapamil, and digoxin for the past 6 months. Her vital signs were: BP 140/80 mmHg, PR 85/min, RR 23/min, T 38°C. She had respiratory distress and central cyanosis. Pulmonary exam showed diffuse wheezing and fine and coarse crackles were heard over the lungs. 

Chest X-ray demonstrated bilateral alveolar consolidation which was more prominent in the lower part of the left lung. Bilateral hilar prominency, mild increase in mediastinal diameter without a gross bone lesion was also apparent.

Arterial blood gas showed: pH 7.42, Pco2 43.9 mmHg, Hco3 27.6, Po2 40.2 mmHg, O2 sat 77.1%. 

Routine laboratory exams including biochemistry tests and complete blood count were normal. Angiotensin converting enzyme (ACE) and rheumatological tests were normal as well, but ESR was elevated to 94 mm in the first hour. Echocardiography showed mildly-reduced left ventricle function with an ejection fraction of 40%, right-sided wall motion abnormality and mild mitral valve regurgitation. 

Doppler sonography of the lower extremity veins did not show evidence of deep vein thrombosis. 

**Figure 1:**
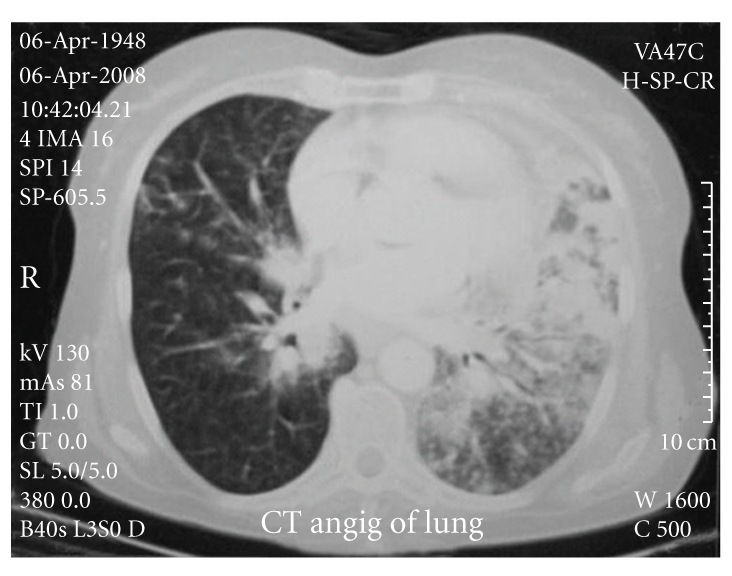
Right-sided parenchymal alveolar consolidation and nodular infiltration in the left lung, nodular opacity in the right middle lobe.

**Figure 2:**
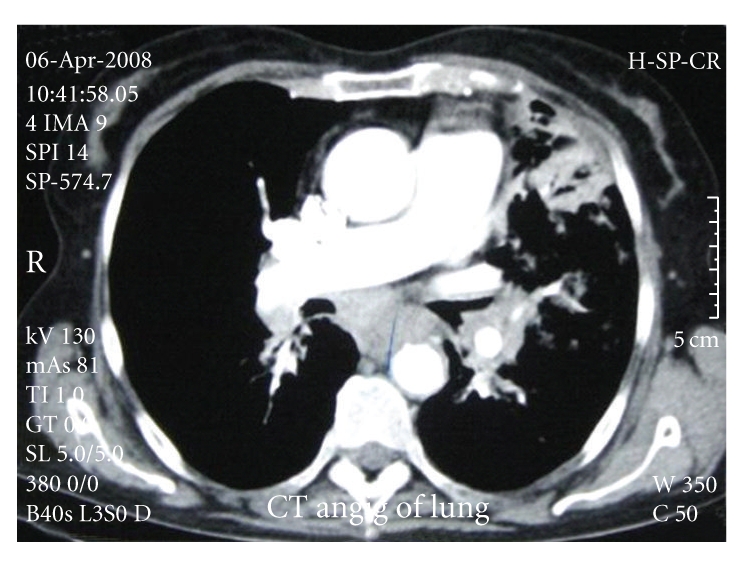
Hilar, mediastinal, and pericardiac lymphadenopathies.

Spiral computed tomography of the thorax with intravenous contrast revealed no intraarterial filling defect, but hilar and mediastinal lymphadenopathies with right-sided parenchymal alveolar consolidation and nodular infiltration were seen in the left lung. Nodular opacity in the right middle lobe with pericardiac lymphadenopathy was also noted. A bleb about one centimeter in diameter was seen in the midzone of the right lung (Figures 1 and 2). 

Bronchoscopy revealed diffuse hemorrhagic erythematous mucosa in the right and left bronchi without an endobronchial lesion. 

**Figure 3:**
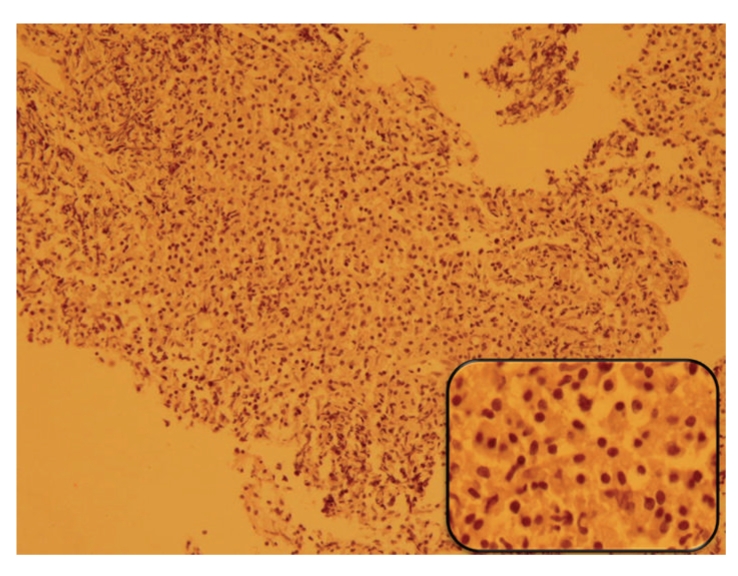
Transbronchial lung biopsy reveals pulmonary parenchyma which is totally replaced by monotonous plasmacytoid cell infiltration (Hematoxylin & Eosin stain).

**Figure 4:**
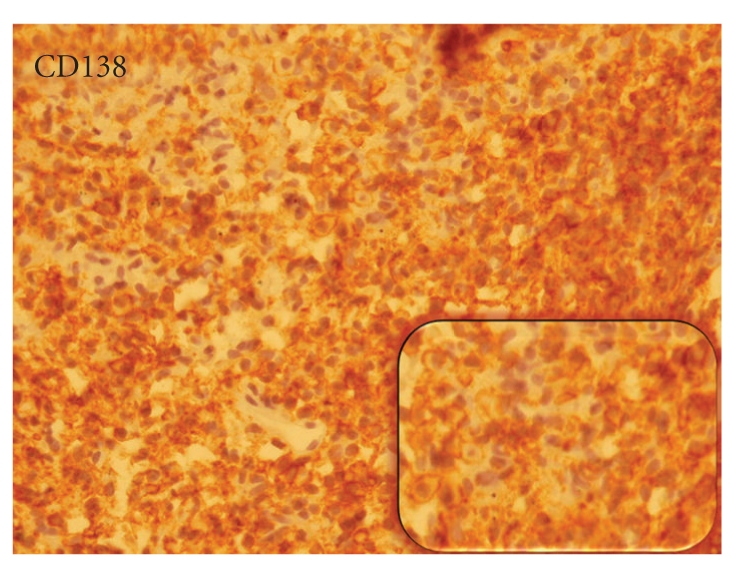
The proliferated cells showing strong positivity for CD138 in Immunohistochemistry staining.

Transbronchial lung biopsy was done and pathologic examination of the specimen demonstrated diffuse plasmacytoid cell proliferation in the alveolar spaces and interstitium. The plasma cells had fine chromatin with mild pleomorphism and mitotic activity without evidence of fibrosis or any other inflammatory cell infiltration. Immunohistochemistry (IHC) revealed diffuse reactivity with CD79a and CD138 and was negative for CD20 and cytokeratin (CK) (Figures 3 and 4). 

Polymerase chain reaction (PCR) analysis of the paraffin embedded block of transbronchial lung biopsy (TBLB) specimen revealed the presence of clonally-rearranged Ig heavy chain gene. According to above findings the diagnosis of plasmacytoma was confirmed and prompt investigation to rule out multiple myeloma was undertaken.

Serum total protein value was 9 gr/dl (normal range: 6.4–8.3 gr/L) with an IgG value of 39.1 gr/L (normal range: 6.58–18.37 gr/L). Levels of other serum immunoglobulins were within normal limit. Serum electrophoresis revealed the M component in the *γ* region. No Bence-Jones protein was detected in urine. Serum calcium and phosphorus were within normal range. Bone survey revealed no abnormality. 

Bone marrow examinations on two occasions were unremarkable with less than 5% plasma cells and no dyscrasia. 

The patient was treated with melphalan and prednisolone. After 4 monthly courses, the chest X-ray became normal and the patient was free of symptoms.

## 3. Discussion

Presence of clonal plasma cell infiltrates in an extramedullary site can be a representation of multiple myeloma or solitary extramedullary plasmacytoma (SEP).

Solitary extramedullary plasmacytoma of the lung is extremely rare, although pulmonary involvement with multiple myeloma is more common [4].

To differentiate solitary EMP from multiple myeloma, bone marrow examination is needed and the patient should have lower than 5% plasma cells with no dyscrasia and a normal skeletal survey [1].

Unlike multiple myeloma, EMPs may not have serum M protein or Bence Jones light chains in urine. However, up to 25% of patients with EMP will show a monoclonal gammopathy (M component). Our patient had M component within the *γ* region on electrophoresis and gene rearrangement study with PCR showed a monoclonal band on electrophoresis.

It is reported that PPP have an equal sex ratio and most of them are asymptomatic [5]. However, symptoms like dyspnea, fever, and hemoptysis have been reported. This case had a pneumonia-like presentation with negative sputum culture. The clinical differential diagnosis included a wide variety of clinical conditions mimicking pneumonia consisting of viral, bacterial, mycobacterial, fungal, and parasitic infections and noninfectious causes such as, organizing pneumonia, hypersensitivity pneumonitis, drug-induced pneumonia, bronchoalveolar carcinoma, collagen vascular diseases, and vasculitis with pulmonary manifestations.

The radiographic presentation of PPP is that of a solitary mass or nodule mostly in hilar areas [5]. Diffuse lung involvement is exceedingly rare and to our knowledge only two cases have been reported in the literature. A case reported by Horiuchi [2] who was a 45-year-old female with fever and diffuse bilateral lung involvement was treated appropriately with melphalan. Another case was reported by Niitsu et al. [3] was a 71-year-old asymptomatic woman with bilateral lung involvement and marked hypergammaglobulinemia. 

The pathologic differential diagnoses include MALT lymphoma with plasma cell differentiation. Extra nodal marginal zone lymphoma of lung has polymorphous microscopic appearance and cellular heterogeneity is a hallmark of these lymphomas. The most characteristic cells are small- to medium-size lymphocytes with irregular nuclear outline (centrocyte-like) and plasma cell differentiation is common. However, other characteristic features including lymphoid follicles with reactive germinal center, lymphoepithelial lesion and fibrosis in addition to varying proportion of plasma cells which are either reactive or neoplastic are also present in involved tissue [4]. IHC findings in MALT lymphoma are CD20^+^, CD79*α* ^+^, CD5^−^, CD10^−^, CD23^−^, and CD43^−^. Our case revealed diffuse monotonous plasma cell infiltration, some with open chromatin pattern, and low mitotic activity without fibrosis or other secondary features which was totally negative for CD20, but diffusely positive for CD138 and CD79a which is consistent with diagnosis of plasmacytoma. Gene rearrangement study on paraffin embedded block specimens via PCR revealed the monoclonal band.

Also, plasmacytoma should be distinguished from plasma cell granulomas in which there is an admixture of inflammatory cells (including lymphocytes and macrophages) within fibrous stroma without evidence of monoclonality by immunohistochemistry or molecular study.

Most of the patients with PPP have been treated with surgery and/or radiotherapy [3]. However, when the pulmonary involvement is diffuse, chemotherapy is the best choice. Two previous cases have been treated with melphalan and prednisolone [2, 3]. The present case also responded well to chemotherapy with melphalan and prednisolone. After 4 monthly courses, chest X-ray became normal and the patient was free of symptoms.

## Abbreviations

ACE: Angiotensin converting enzyme
ALT: Alanine aminotransferase
AST: Aspartate aminotransferase
BP: Blood pressure
CBC: Complete blood count
CT-scan: Computed Tomography scanning
ESR: Erythrocyte sedimentation rate
FBS: Fasting blood sugar
LDH: Lactate dehydrogenase
PR: Pulse rate
RR: Respiratory rate
T: Temperature.
